# Point-of-Care Ultrasound in Nephrology: Beyond Kidney Ultrasound

**DOI:** 10.3390/diagnostics15030297

**Published:** 2025-01-27

**Authors:** Victor Hugo Gómez-Johnson, Salvador López-Gil, Eduardo R. Argaiz, Abhilash Koratala

**Affiliations:** 1 Nephrology Department, Instituto Nacional de Cardiología, Ignacio Chávez, Mexico City 14080, Mexico; vgomezjohnson@gmail.com (V.H.G.-J.); salvadorlgil@gmail.com (S.L.-G.); 2 Tecnológico de Monterrey, Escuela de Medicina y Ciencias de la Salud, Mexico City 64710, Mexico; lalo.argaiz@gmail.com; 3 Departamento de Nefrología y Metabolismo Mineral, Instituto Nacional de Ciencias Médicas y Nutrición Salvador Zubirán, Mexico City 14080, Mexico; 4 Division of Nephrology, Medical College of Wisconsin, Watertown Plank Rd., Milwaukee, WI 53226, USA

**Keywords:** POCUS, point-of-care ultrasound, AKI, acute kidney injury, nephrology

## Abstract

Point-of-care ultrasound (POCUS) has increasingly become an integral part of clinical practice, particularly in nephrology, where its use extends beyond renal assessment to include multi-organ evaluations. Despite challenges such as limited ultrasound training and equipment access, especially in low- and middle-income countries, the adoption of POCUS is steadily rising. This narrative review explores the growing role of multi-organ POCUS in nephrology, with applications ranging from the assessment of congestion phenotypes, cardiorenal syndrome, and hemodynamic acute kidney injury (AKI) to the evaluation of arteriovenous fistulas and electrolyte disorders. In nephrology, POCUS enhances clinical decision making by enabling rapid, bedside evaluations of fluid status, cardiac function, and arteriovenous access. Studies have demonstrated its utility in diagnosing and managing complications such as heart failure, cirrhosis, and volume overload in end-stage renal disease. Additionally, POCUS has proven valuable in assessing hemodynamic alterations that contribute to AKI, particularly in patients with heart failure, cirrhosis, and systemic congestion. This review highlights how integrating ultrasound techniques, including lung ultrasound, venous Doppler, and focused cardiac ultrasound, can guide fluid management and improve patient outcomes. With advancements in ultrasound technology, particularly affordable handheld devices, and the expansion of targeted training programs, the potential for POCUS to become a global standard tool in nephrology continues to grow, enabling improved care in diverse clinical settings.

## 1. Background

The utilization of point-of-care ultrasound (POCUS) as a fifth pillar of physical examination has markedly increased in recent years. Despite the uneven global availability of ultrasound technology, it is encouraging to see a growing number of healthcare professionals across various specialties adopting POCUS. This trend reflects the perceived need for enhanced diagnostic capabilities to facilitate more informed treatment decisions at the bedside [1].

For nearly a decade, insufficient training and limited access to ultrasound equipment have been recognized as the two primary barriers to the widespread implementation of POCUS. While the training issue is being addressed in countries like the United States, where over 70% of medical schools have integrated ultrasound training into their curricula [2], developing nations still face significant challenges. The second barrier, involving equipment availability, is more challenging to address. A recent study revealed that the availability of ultrasound machines in low- and middle-income countries is approximately 2% [3,4]. The emergence of affordable handheld ultrasound devices offers a promising opportunity to help bridge this gap to some extent.

In nephrology, multi-organ POCUS is increasingly being integrated into routine practice, enabling the rapid assessment of diverse causes of acute kidney injury (AKI), such as urinary obstruction [5] and hemodynamic aberrations like congestive, distributive, obstructive, or low cardiac output (CO) disorders [6,7,8,9]. A recent position statement from the International Alliance for POCUS in Nephrology highlights the scope of POCUS in this specialty, focusing on multi-organ applications beyond renal ultrasound [10]. These applications can serve for diagnostic purposes, narrowing the differential diagnosis, or guiding treatment in different scenarios common to the practice of nephrology.

It is important to establish that, despite its rapid adoption, POCUS is highly operator-dependent, and there is a pressing need for developing an appropriate training program that can ensure a minimum level of competency to minimize missed findings, the misinterpretation of findings, errors, or the misidentification of structures. Additionally, it should be noted that POCUS is not a substitute for conventional imaging methods and radiological studies, which must be obtained when indicated. This narrative review explores various clinical scenarios where multi-organ POCUS serves as a valuable tool for nephrologists. Each section provides a theoretical background, outlines relevant ultrasound applications, and highlights evidence supporting its utility.

## 2. Assessment of Congestion Phenotypes

Assessing hemodynamic status and congestion is essential for nephrologists, who regularly manage complex fluid and electrolyte disorders. Recent reviews have categorized congestion into two primary phenotypes: intravascular congestion and tissue congestion. Intravascular congestion primarily refers to an increase in central pressures, which may occur due to an inability to mobilize the total blood volume in patients with reduced cardiac function or an endogenous increase in preload. This preload increase is mediated by an augmented venous tone, resulting from hyperactivation of the sympathetic nervous system (SNS). This hyperactivation recruits unstressed volume mainly in the splanchnic circulation, alongside impaired storage venous capacity [11,12]. Tissue congestion, by contrast, occurs when capillary hydrostatic pressure surpasses the lymphatic drainage capacity. This is mainly driven by elevated central venous pressure, which hinders lymphatic drainage in patients with heart failure, and by compromised vascular integrity. The ultimate result is an increase in interstitial fluid volume, manifesting as pleural effusion, ascites, and edema, among other symptoms [12]. Traditional assessments rely on clinical findings such as edema, jugular venous distention, hepatojugular reflux, and rales, among others. Over time, laboratory studies have been incorporated to better categorize congestion phenotypes. For instance, Carbohidrate Antigen 125 (CA-125) more accurately reflects tissue congestion, whereas NT-proBNP (N-terminal pro b-type natriuretic peptide) offers greater insight into intravascular congestion [13].

**Figure 1:**
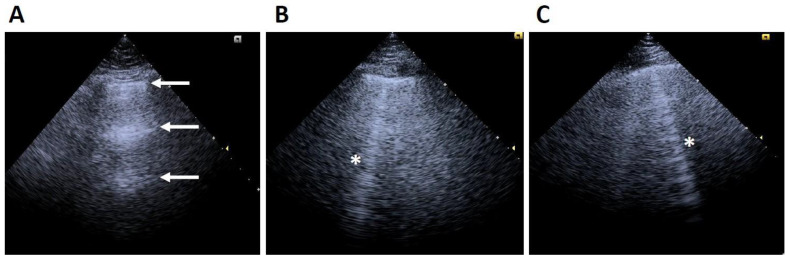
(**A**) Normal lung ultrasound. Arrows point to normal (**A**) lines. (**B**,**C**) Comet tail artifacts or “B-lines” (*).

Lung ultrasound (LUS) is a widely recognized POCUS technique for accurately assessing and quantifying extravascular lung water (EVLW) by identifying sonographic artifacts linked to fluid accumulation in the lung interstitium and alveoli. In pulmonary edema, fluid buildup thickens the interlobular septa and alters the lung interface’s acoustic properties, producing a distinctive B-line pattern. These B-lines are vertical, hyperechoic artifacts that originate at the pleural line, extend to the bottom of the ultrasound field without fading, and move synchronously with pleural sliding. They are categorized as *ringdown* artifacts, where ultrasound waves repeatedly reflect between fluid-filled interlobular septa and adjacent alveolar walls, creating bright, linear echoes [14] (Figure 1).

LUS has been shown to be considerably more sensitive than auscultation or even chest radiography in detecting pulmonary edema [15,16]. The addition of LUS use in the emergency department can help differentiate between various etiologies of dyspnea and reduce the time to diagnosis and decision making by up to 31 min [17]. Several interventional trials have been performed using LUS in patients with acute and chronic heart failure and have shown to reduce the number of emergency department visits [18,19,20]. Therefore, LUS is now becoming a fundamental tool for evaluating patients with suspected heart failure. This is particularly significant for nephrologists, given the high prevalence of heart failure among individuals with chronic kidney disease. Additional considerations for patients undergoing chronic hemodialysis are discussed later.

There are important caveats of LUS in the assessment of pulmonary congestion. The most relevant limitation is that B-lines are not specific to cardiogenic pulmonary edema and can be seen in patients with interstitial pneumonia (such as viral pneumonia) and several forms of interstitial lung disease. When present, findings such as subpleural consolidations, pleural thickening, and irregularities can suggest a diagnosis of primary lung disease [21]. However, complementing LUS with the assessment of left-sided cardiac filling pressures on focused cardiac ultrasound can increase the specificity of this examination. Left-sided filling pressures can be estimated using trans-mitral pulsed-wave Doppler (E/E’ ratio) and peak tricuspid regurgitation velocity (TRVmax) [22]. Both the E/E’ ratio and TRVmax require advanced POCUS skills but, with enough training, can be performed at the bedside by the nephrologist. E/E’ is obtained by placing the Doppler sample volume at the mitral valve opening to obtain the trans-mitral waveform. The maximum velocity of flow during passive left ventricular (LV) filling is then measured (E wave). The velocity of the E wave depends on two main variables: LV relaxation and left atrial pressure (LAP). Both increased LV relaxation and increased left atrial pressure will result in a larger E wave. Thus, the E wave by itself cannot distinguish between increased LAP or hyperdynamic LV. Because of this, myocardial relaxation velocity should also be obtained using tissue Doppler at the septal and lateral mitral annulus during ventricular relaxation (E’ wave). The ratio of E to E’ thus accounts for myocardial relaxation and is a surrogate of LAP [23]. TRVmax is acquired using continuous-wave Doppler aligned to the tricuspid regurgitation jet, which can be found using color Doppler of the tricuspid vale. An elevated TRVmax (>2.8 m/s) results from a high-pressure gradient between the right ventricle (RV) and the RA during systole, which occurs when the pulmonary artery’s systolic pressure is elevated [24].

**Figure 2:**
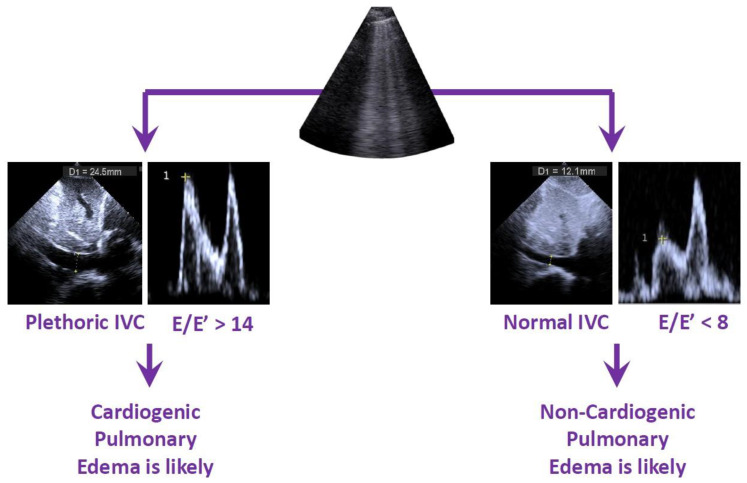
Combined lung ultrasound and focused cardiac ultrasound. An algorithm to determine the likelihood of cardiogenic vs. non-cardiogenic pulmonary edema based on assessing cardiac filling pressures is presented. Note: E/E’ values between 8–14 are considered indeterminate. Tissue Doppler (E’) tracing is not depicted in the illustration.

While these techniques require advanced ultrasound skills, it is important to realize that, in patients with left heart failure, right atrial pressure increases proportionally to increasing left-sided filling pressures [25]. Hence, right atrial pressure provides a measure of the filling pressure of the whole heart [26]. POCUS evaluation of right atrial (RAP) pressure is relatively straightforward and can be performed using ultrasound of the inferior vena cava (IVC) or internal jugular vein (IJV). The likelihood of cardiogenic pulmonary edema in a patient with B-lines on LUS increases when ultrasonographic evidence of elevated right atrial pressure is also present (Figure 2). It is our opinion that cardiology consultation should be considered for any new abnormal finding on POCUS, including elevation in left- or right-sided filing pressures, even with no appearing structural cardiac abnormalities.

## 3. Assessment of Cardiorenal Syndrome

It is now well established that increased RAP is the main pathophysiological mechanism through which patients with acute decompensated heart failure (ADHF) develop AKI [27]. However, although less frequent, renal dysfunction in the setting of heart failure can also be related to decreased CO secondary to pump failure. The term “cardiorenal” syndrome is thus an umbrella term encompassing the different hemodynamic contributors to AKI in this setting [28].

POCUS evaluation is becoming an essential bedside tool for phenotyping the primary hemodynamic changes that contribute to AKI. Focused cardiac ultrasound (FoCUS) plays a key role in identifying patients with low CO or cardiogenic shock. Findings such as a severely reduced ejection fraction, right ventricular failure, or severe valvular disease can heighten the likelihood of this condition [29]. Additionally, Doppler assessment provides an accurate estimation of CO. This involves measuring the left ventricular outflow tract diameter and velocity–time integral (VTI) in the parasternal long-axis and apical five-chamber views, respectively. Stroke volume is then calculated using the formula left ventricular outflow tract (LVOT) area (πr^2^) × VTI, and when multiplied by the heart rate, this gives the CO: (CO = stroke volume × heart rate) [30,31].

RV failure is a common occurrence in cardiorenal syndrome as the underlying cause of elevated RAP. RV failure can be assessed using POCUS by observing RV dilation, interventricular septal flattening, and RV systolic dysfunction [32]. RV dilation can be identified in the parasternal long axis by comparing the size of the RV with that of the left atrium and ascending aorta; normally, these three chambers should be roughly the same size. The RV should not exceed two-thirds of the size of the LV in an apical four-chamber view. RV systolic function can be assessed by measuring the tricuspid annular plane systolic excursion (TAPSE) using M-mode. TAPSE is a straightforward measurement that correlates well with the RV ejection fraction as determined by magnetic resonance imaging (MRI) [33]. RV dilation can also lead to functional tricuspid regurgitation (TR), which has recently garnered attention due to its association with hepatic and renal congestion [34]. The presence of TR and its severity can be qualitatively assessed using color Doppler across the valve [35].

IVC ultrasound, which measures its diameter and respiratory collapse, remains the most common POCUS parameter for estimating RAP. However, it is crucial to understand that IVC size and collapsibility are influenced by factors other than RAP. For instance, increased intra-abdominal pressure or vigorous respiratory effort can cause the IVC to collapse, even when RAP is significantly elevated [36,37]. Another important caveat of IVC ultrasound is that its diameter is usually assessed only on its long axis, while this is a three-dimensional structure. Moreover, in some patients, IVC collapse can be more pronounced in the latero-lateral plane. Observing the IVC on its short axis allows for the measuring of its area and is a more accurate way to assess RAP [38,39]. Additionally, several studies have demonstrated that POCUS of the internal jugular vein (IJV), to assess the height of the fluid column, can more accurately and reliably identify elevated RAP, even outperforming IVC POCUS in certain conditions such as cirrhosis [40,41,42]. Limitations of IJV POCUS include thrombosis of the vessel, venous stenosis (frequently occurring in patients with end-stage renal disease and multiple central venous catheters), and artifacts arising from improper neck positioning [43]. Additionally, recent publications have shown that IVC ultrasound together with venous Doppler to interrogate the flow pattern of the hepatic, portal, and intra-renal veins can estimate RAP with more accuracy than IVC alone [44,45].

**Figure 3:**
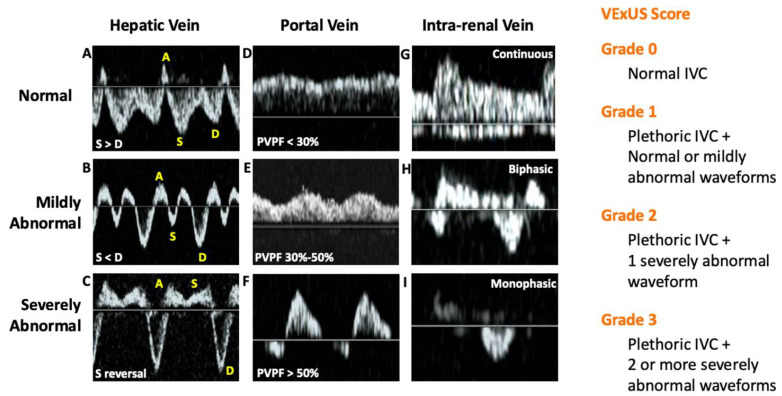
Left: Representative waveforms of normal and abnormal hepatic (HVD), portal (PVD), and intra-renal venous Doppler (IRVD). (**A**) Normal HVD, note S wave > D wave; (**B**) moderately abnormal HVD, note S wave < D wave; (**C**) severely abnormal HVD, note reversed S wave; (**D**) normal PVD, pulsatility fraction < 30%; (**E**) moderately abnormal PVD, pulsatility fraction between 30 and 50%; (**F**) severely abnormal PVD, pulsatility fraction >50%; (**G**) normal IRVD, continuous pattern; (**H**) moderately abnormal IRVD, biphasic pattern, and (**I**) severely abnormal IRVD, monophasic pattern. Right: VExUS grading system.

The combination of increased RAP and a maximally distended peripheral venous system results in an inability to accommodate retrograde flow [46]. When this happens, retrograde atrial flow can reach the venules of peripheral organs, potentially leading to organ congestion [47]. Retrograde atrial flow can be assessed at different organ sites such as hepatic, portal, and intra-renal vessels using venous Doppler. Recently, a grading system for quantifying altered venous flow to these organs has been developed and named the venous excess ultrasound score (VExUS) [48]. VExUS involves measurements of the IVC and the flow pattern in the hepatic vein (HV), portal vein (PV) and intra-renal veins (IRVs). The HV, being the first conduit from the right atrium (RA), exhibits waveforms like the central venous pulse. The HV has four waves: wave “A” (atrial systole), wave “S” (ventricular systole), wave “V” (atrial filling), and wave “D” (ventricular diastole). As RAP increases, the dominance of wave S diminishes and may merge with wave A, producing S wave reversal, observed in severe congestion states [49]. The portal vein is notable for its easy acquisition and interpretation. Hepatic sinusoids buffer the increase in RAP, resulting in a monophasic non-pulsatile flow. However, when RAP exceeds this buffering capacity, pulsatility increases, calculated by the portal vein pulsatility fraction (Vmax − Vmin / Vmax × 100), with values of 30–50% indicating moderate congestion and >50% severe congestion [50]. Lastly, the intra-renal venous Doppler (IRVD) categorizes flow into continuous, biphasic, and monophasic according to RAP increase [51]. Recent studies have demonstrated a correlation between IRVD severity and prognosis and mortality in patients with ADHF [52,53]. IRVD is the most challenging waveform to acquire; however, a recent study demonstrated that intra-renal and portal venous Doppler provide very similar information in patients with pulmonary hypertension [54]. The VExUS score was originally validated in a cohort of patients undergoing cardiac surgery. This study showed that an altered VExUS score was the most important predictor of AKI in this population, outperforming invasive cardiac pressure measurements [48]. The VExUS grading system is summarized in Figure 3.

The physiological rationale for using VExUS in patients with cardiorenal syndrome was recently highlighted in a prospective study involving 77 patients with acute coronary syndrome (ACS). In this study, 100% of patients with VExUS grade 3 developed AKI. VExUS grades greater than 1 were significantly associated with an increased risk of AKI (OR 6.15, 95% CI 1.26–29.9, and *p* = 0.02), and a better area under the curve (AUC) of 0.787 compared to the cardiac index (CI) with an AUC of 0.629 [55]. Additionally, VExUS grade 3 was significantly associated with higher in-hospital mortality in patients with heart failure (OR 8.03, 95% CI 2.25–28.61, and *p* = 0.001) [56]. Regarding diuretic guidance in this patient population, the approach remains to be fully elucidated. A pilot randomized trial using VExUS as a target for decongestion in cardiorenal syndrome showed greater odds to achieve decongestion in the VExUS-guided group [57]. Currently, a single-center, double-blind, randomized controlled trial is being conducted to address this question (NCT06065163).

## 4. Assessing Hemodynamic AKI in Patients with Cirrhosis

In advanced cirrhosis, hyperactivation of the neurohumoral axis, primarily represented by the SNS, the renin–angiotensin–aldosterone system (RAAS), and the consequent release of arginine vasopressin (AVP) occurs in response to excessive vasodilation of the splanchnic circulation. This vasodilation, mainly mediated by nitric oxide (NO) due to structural hepatic damage, is one of the key hemodynamic derangements in these patients [58]. Renal retention of salt and water increases the extracellular volume, leading to a compensatory increase in CO [59]. Thus, in compensated cirrhosis, renal perfusion is maintained. However, compensation is fragile, and any additional hemodynamic insult such as sepsis or hypovolemia can result in AKI. Importantly, patients with cirrhosis have a very high prevalence of diastolic disfunction, often called cirrhotic cardiomyopathy [60].

The compensatory increase in extracellular volume needed to compensate for splanchnic vasodilation can have deleterious consequences if myocardial relaxation is severely abnormal, resulting in a suboptimal increases in CO and increased cardiac filling pressures [61]. At this point, further resuscitative efforts with volume expansion will only result in worsening heart failure and hepato-cardio-renal syndrome [62]. Thus, while splanchnic vasodilation is the main hemodynamic alteration in cirrhosis, many additional insults can contribute to AKI in these patients [63,64].

POCUS can play a key role in diagnosing and managing hepatorenal syndrome. Before administering empiric albumin, it is essential to assess pulmonary and systemic congestion to determine *volume tolerance*. If B-lines are detected, FoCUS should be performed to evaluate cardiac filling pressures, including the E/A ratio, E/e’ ratio, LAVI, and TRVmax for left-sided pressures, and/or IVC or IJV POCUS for RAP. Further investigations may include intra-renal venous Doppler to assess end-organ congestion. Portal and hepatic vein Doppler can be unreliable, as chronic liver disease may influence their interpretation [65,66]. Alterations in these parameters suggest underlying congestive heart failure, and volume resuscitation is discouraged. If venous congestion is severe, decongestive treatment with diuretics is suggested as the predominant contributor to AKI is likely to be type 1 cardiorenal syndrome [67]. In fact, an elegant study invasively assessed cardiac filling pressures and found that patients presenting with increased pulmonary artery wedge pressure show improved renal function after diuresis [68].

The absence of venous and pulmonary congestion is frequently interpreted as a sign of hypovolemia [69]. This is a misconception, and the correct interpretation is that heart failure is not present regardless of volume status. Even an over-resuscitated hypervolemic patient will not necessarily develop an increase in cardiac filling pressures if systolic and diastolic function are normal. If clinical situation suggests the possibility of hypovolemia (history of fluid loss), then a normal IVC/IJV and LUS can reassure the clinician that the patient is *fluid-tolerant* and volume administration is worth trying [70]. Finding a low CO by evaluating LVOT diameter and LVOT VTI supports the diagnosis of hypovolemia; however, it is important to note that vasodilatory states such as sepsis can have low CO and exhibit sonographic similarity to true volume depletion [70].

Various studies have been published that address different POCUS applications specifically in patients with cirrhosis. IVC ultrasound has shown moderate correlation with RAP in this population [71]. A couple of studies have found that between 21 and 45% of patients with cirrhosis presenting with AKI have a plethoric IVC at presentation [72,73], suggesting underlying congestive heart failure. In both observational studies, authors recommended against fluid expansion and suggested initiating diuretic treatment. However, altered liver and vascular anatomy in cirrhosis can alter IVC interpretation. The stiff parenchyma surrounding the IVC can cause a fixed diameter in a considerable number of patients [74]. Additionally, collateral venous circulation can increase IVC volume and result in a dilated vessel even with normal RAP [75]. Because of these issues, IJV POCUS has been proposed as an alternative way to assess RAP in patients with cirrhosis, showing better accuracy and easier acquisition [76]. As stated previously, multi-organ POCUS provides a more complete evaluation by assessing left- and right-sided filling pressures and systolic function. Severe diastolic dysfunction on FoCUS has been associated with lack of response to terlipressin in these patients [77]. Most patients with cirrhosis and venous congestion have left ventricular diastolic dysfunction as the underlying cause; however, porto-pulmonary hypertension and dilated cardiomyopathy should be ruled out.

While these recommendations are not currently cited in international consensus guidelines, it is noteworthy that the routine administration of albumin is no longer recommended, according to the recent update from the International Club of Ascites (ICA) and the Acute Disease Quality Initiative (ADQI). These guidelines emphasize that intravascular volume assessment should be performed, and if congestion is present, albumin administration should be avoided as it delays the administration of terlipressin [59].

## 5. Arteriovenous Fistula Assessment

**Figure 4:**
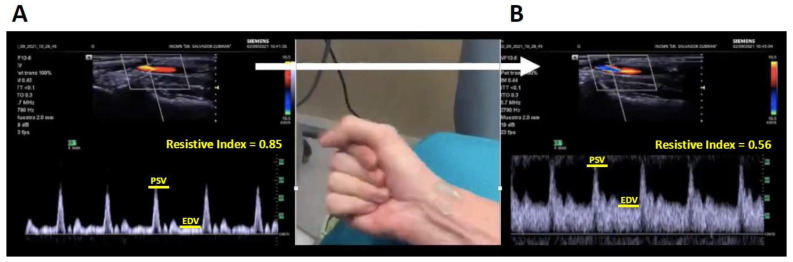
Pulsed-wave Doppler image of radial artery: (**A**) Doppler signal at baseline and (**B**) Doppler signal after a maneuver to recruit blood flow to the hand muscles. PSV = peak systolic velocity; and EDV = end-diastolic velocity. The resistive index is calculated as (PSV − EDV)/PSV.

Physical examination is essential for evaluating arteriovenous fistulas (AVFs) in patients undergoing renal replacement therapy (RRT). Nephrologists must be adept at detecting inflow and outflow stenosis by assessing thrill, bruit, augmentation, pulsatility, and collapse upon arm elevation [78]. However, POCUS significantly enhances the thoroughness of this assessment. Examining the patient even before fistula creation can help predict its maturation. While the latest Kidney Disease Outcomes Quality Initiative (KDOQI) vascular access guidelines no longer recommend routine pre-creation mapping for all patients, it is still advised for high-risk individuals, such as the elderly, females, and those with a history of central venous catheters (CVCs), peripheral lines, cardiac pacemakers, and other comorbidities [79]. Mapping should be performed by a trained and experienced nephrologist, ideally using a spectral Doppler. This may or may not fall within the scope of POCUS depending on the geographic variations in practice. Venous evaluation of the upper extremity should include both superficial (cephalic and basilic) and deep veins (radial and ulnar), focusing on patency, stenosis sites, and diameters greater than 2 mm [80]. Arterial assessment includes the evaluation of the brachial, ulnar, and radial artery diameters and should include the pulsed-wave Doppler assessment of flow pattern. The normal flow pattern is described as “triphasic”, with little diastolic flow. Alternating between clenching the fist and fully opening the hand can recruit blood flow to the hand muscles and increase diastolic blood flow, signifying sufficient arterial reserve. A decrease in the resistive index to below 0.7 is associated with a higher likelihood of fistula maturation [81] (Figure 4). A modified Allen test looking at changes in palmar blood flow with radial artery compression has been described and can assess the presence of collateral circulation to the hand [82].

Once the AVF is created, B-mode ultrasound can be used to assess both the course and depth of the fistula. If the depth exceeds 6 mm, cannulation will be challenging and impossible in certain cases [83]. Additionally, B-mode ultrasound can detect the presence of aneurysms or hematomas around the fistula. Assessment of AVF flow is considered an advanced POCUS application, and it can be assessed by measuring the area and the mean velocity of flow in the brachial artery. Multiplying the velocity in cm/min × the area in cm^2^ will result in flow cm^3^/min [83].

Finally, hemodynamic assessment is critical following the creation of an AVF. The creation of the vascular is followed by a drop in systemic vascular resistance and blood pressure while venous return and left ventricular end-diastolic volume (LVEDV) increase, resulting in elevated CO. In some patients, a persistently elevated CO will lead to significantly increased cardiac filling pressures [84,85]. Thus, using POCUS for patients with AVF can also help identify those with heart failure caused by a high-flow AVF. Worsened right ventricular dilation or function following the creation of an AVF has been associated with increased mortality [86].

## 6. Electrolyte Disorders

Hyponatremia remains one of the most common electrolyte disorders managed by nephrologists. Since hemodynamically mediated non-osmotic antidiuretic hormone (ADH) secretion is a frequent cause of hyponatremia [87], POCUS can complement and enhance the physical examination in these cases. The growing number of reports on nephrologists using POCUS to assess the hemodynamic changes contributing to hyponatremia reflects increased adoption of this technique. In these studies, POCUS has successfully identified issues such as over-diuresis in ADHF, under-resuscitation in hypovolemia, and persistent venous congestion [88,89,90].

In a recent study, 19 patients with severe hyponatremia (<120 mmol/L) were evaluated using both clinical and ultrasonographic assessments conducted independently. The goal was to assess the percentage correlation with the final diagnosis (hypo-, hyper-, or euvolemia). POCUS correlated with 84% of the diagnoses, compared to physical examination alone, which correlated with 63% [91]. The integration of POCUS in evaluating hyponatremia does not replace physical examination but can significantly enhance diagnostic accuracy when interpreted in the clinical context.

## 7. Renal Replacement Therapy

Volume overload in end-stage renal disease (ESRD) has been linked to increased mortality, making it essential for clinical assessments to be supplemented with additional tools to guide fluid removal during hemodialysis sessions [92]. Bioimpedance has long been used to measure extracellular water in these patients; however, recent studies have highlighted ultrasound as a promising method for this patient group [93]. The prevalence of heart failure with preserved ejection fraction (HFpEF) is particularly high among patients receiving RRT, making the estimation of filling pressures crucial [94]. Extensive studies have evaluated the use of LUS, with findings from Zoccali et al. demonstrating that, despite the lack of strong outcomes related to guided fluid removal, fewer hospitalizations due to heart failure decompensation were observed [95]. Additionally, the assessment of intravascular congestion through the VExUS score has gained attention in the academic literature as a dynamic method for evaluating fluid extraction, though robust evidence for its daily use is still lacking [96,97]. Recent studies suggest that IVC diameter is a sensitive tool for assessing volume removal as it outperformed central venous pressure in tracking ultrafiltration volume [98].

Finally, the prevalence of pericardial effusion is critically important in patients with ESRD. A recent study revealed that up to 75% of patients listed for kidney transplantation had pericardial effusion, with 17% exhibiting signs of cardiac tamponade [99]. Consequently, using ultrasound to identify early signs, such as systolic collapse of the right atrium, and late signs, such as diastolic collapse of the right ventricle and tricuspid and mitral valve flow variation with pulsed-wave Doppler, is essential for recognizing this serious condition [100].

In conclusion, for patients undergoing RRT, it is imperative to utilize a broad array of tools rather than rely solely on traditional dry-weight estimates. As our understanding of pathophysiology improves, incorporating ultrasound techniques can significantly enhance patient care.

## 8. Future Directions and Conclusions

The use of POCUS has become a daily tool in nephrology and has seen exponential growth. However, it is still not part of the educational curriculum for this specialty in many parts of the world. It has been just over five years since early publications on POCUS in nephrology highlighted its importance in addressing bedside questions ranging from urinary retention to pericardial effusion [101]. Today, there are compelling proposals for incorporating this tool into nephrology training programs. Different degrees of competency in POCUS can be pursued: for example, renal and bladder ultrasound, LUS, IVC, IJV, and basic cardiac windows can be considered basic ultrasound applications, while E/E’, TRVmax, venous Doppler, and AVF flow assessment represent advanced applications.

While various barriers remain, they are gradually being overcome due to a strong desire for learning and the dedicated efforts of experts who have fostered the growth of this field. As a result, we believe that, with time and continued effort, multi-organ POCUS will become a widely established tool worldwide.
